# Repeatability of breast density visual assessment

**DOI:** 10.1186/bcr3282

**Published:** 2012-11-09

**Authors:** L Walshaw, JC Sergeant, M Wilson, S Steed, N Barr, U Beetles, C Boggis, S Bundred, S Gadde, Y Lim, S Whiteside, DG Evans, A Howell, SM Astley

**Affiliations:** 1 Manchester Medical School, University of Manchester, UK; 2 Institute of Population Health, University of Manchester, UK; 3 Nightingale Centre and Genesis Prevention Centre, University Hospital of South Manchester, Manchester, UK; 4 Department of Medical Statistics, University Hospital of South Manchester, Manchester, UK

## Introduction

Breast density, measured as the proportion of the breast occupied by fibroglandular tissue in a mammogram, is a strong and modifiable risk factor for breast cancer. Area-based estimates made by expert observers are a practical approach, but are subjective. Here we investigate repeatability of visual assessment of percentage breast density.

## Methods

Seven mammographic film readers re-assessed the density of 100 normal full-field digital mammogram cases for which they had made density estimates at least 1 year previously as part of the Predicting Risk of Cancer at Screening (PROCAS) study. The mammograms for a given reader were selected to show a range in density, by randomly sampling 10 cases from each decile of density assessed by that reader. They were reviewed in similar reading conditions on both occasions using a visual analogue scale to record the assessments.

## Results

For the majority of readers the difference in mean density between the two sets of readings was less than 6%, but the largest discrepancy between means was 14.7%. Bland-Altman plots were generated for each reader and showed considerable variation between readings on the two occasions. At best, the limits of agreement were -12.46% to +17.02%, and at worst they were -14.50% to +40.98%. The largest difference between first and second readings for each reader ranged from 26 to 65%.

## Conclusion

Although density estimates made by a subset of these readers have been strongly related to cancer risk, the variability in reproducibility calls into question the usefulness of subjective assessment without prior evaluation of reader performance.

